# Herpesviruses and human papillomaviruses in saliva and biopsies of patients with orofacial tumors

**DOI:** 10.1016/j.clinsp.2024.100477

**Published:** 2024-08-31

**Authors:** Paa-Kwesi Blankson, Grace E. Parkins, Harriet Naa Afia Blankson, Abiodun Olubayo Fasola, Prince J. Pappoe-Ashong, Matthew O. Boamah, Richard Harry Asmah

**Affiliations:** a Department of Oral and Maxillofacial Surgery, Dental School, University of Ghana/Oral and Maxillofacial Surgery Unit, Korle-Bu Teaching Hospital, Accra, Ghana; b School of Biomedical and Allied Health Sciences, College of Health Sciences, University of Ghana, Korle-Bu, Accra, Ghana; c Research Center Borstel, Borstel, Germany; d Department of Oral and Maxillofacial Surgery, University College Hospital, Ibadan, Nigeria; e Virology Unit, Department of Medical Microbiology, School of Medicine, University of Ghana, Ghana; f Department of Biomedical Sciences, School of Basic and Biomedical Sciences, University of Health and Allied Health Sciences, Ho, Ghana

**Keywords:** Herpesvirus, HPV, Orofacial tumor, Length-of-stay, Ghana

## Abstract

**Objectives:**

To determine the prevalence and association of HPV and Herpesviruses in saliva and tissue samples of patients with orofacial tumors.

**Methods:**

Biopsies of tumors were done, and saliva samples were collected from patients with orofacial tumors for the determination of viruses using nested multiplex PCR. Independent variables were sex, age, comorbidities, tumor stage, and length of stay. Outcome variables were the presence or absence of herpesviruses and HPV. Descriptive summaries and inferential statistics were done.

**Results:**

A hundred patients were included in the study. Prevalence of herpesviruses and HPV were 17.6 % and 57.0 % in tumors, and 48.3 % and 60.0 % in the saliva of patients respectively. Herpesviruses detected included EBV (21.3 %), HHV-7 (11.2 %), CMV (6.7 %), HSV-1 (5.1 %), HSV-2 (1.1 %), VZV (1.1 %), and Kaposi sarcoma virus (0.6 %). The most prevalent HPV genotypes were HPV-42 (29 %), HPV-43 (22.7 %), HPV-52 (22.2 %), HPV-39 (18.8 %), and HPV-18 (9.1 %). The odds of EBV being detected in malignant orofacial tumors were 2 times that of benign orofacial tumors. HPV DNA in the saliva of patients with orofacial tumors was 69.7 %, compared to 18.2 % of the control sample (p < 0.001). The median length of stay for all participants was 6.5 days, those associated with viruses stayed longer.

**Conclusion:**

There was a high prevalence of Herpesviruses and HPV in saliva and tumor samples of patients with orofacial tumors, signalling some potential for more work to be done in this area.

## Highlights

• Herpesviruses and various HPV genotypes are detectable in tumor samples and saliva of patients with orofacial tumors.
• Prevalent genotypes were EBV (21.3 %), HPV-42 (29 %), HPV-43 (22.7 %), HPV-52 (22.2 %), HPV-39 (18.8 %), and HPV-18 (9.1 %).
• Herpesvirus and HPV DNA were significantly more in the saliva of patients with orofacial tumors than in the control population.
• Epstein Barr virus was more likely to be found in malignant tumor samples than benign orofacial tumors.

## Introduction

Benign and malignant orofacial tumors are essential clinical entities associated with considerable morbidity and mortality. As the incidence of oral cancer is noted to be rising, orofacial tumors, particularly oral cancers are becoming crucial public health issues in recent years.^1^

While oral cancers have been associated with tobacco smoking and chewing, alcohol, and betel quid use,^2^ both benign and malignant orofacial tumors have also been suggested to be related to the Human Papillomavirus (HPV), and Herpesviruses.^3^^,^^4^ Aside being heavily implicated in the etiology of oral cancers, some authors have also documented a possible relationship between HPV and Ameloblastoma.^5^ The role of Herpesviruses in the pathogenesis of oral cancer has been suggested in the literature for the past several decades,^6^ and recent studies continue to implicate herpesviruses in pre-cancerous and cancerous lesions, though the mechanism of oncogenesis is not clearly agreed on.^4^ For HPV however, substantial research has demonstrated that they induce and maintain the malignant phenotype through the E6 and E7 proteins.^7^ The E6 protein can specifically bind to the cellular p53 protein, with the resultant breakdown of p53 and loss of its concentration in cancer cells.^8^ The results of this are numerous, including the lack of DNA repair following damage by other agents and the lack of apoptosis ability.

Though more than 130 herpes viruses are known, only nine sub-types are known to infect humans.^9^ These are Herpes Simplex Viruses 1 and 2 (HSV-1 and HSV-2), Varicella-Zoster Virus (HHV-3), Epstein-Barr virus (EBV or HHV-4), human cytomegalovirus (HCMV or HHV-5), Human Herpesvirus 6A and 6B (HHV-6A and HHV-6B), Human Herpesvirus-7 (HHV-7), and Kaposi's sarcoma-associated Herpesvirus (HHV-8).^9^ The HPV family also consists of more than 200 genotypes, classified by their ability to infect and transform epithelial cells.^10^

Incidentally, Sub-Saharan Africa (SSA) is reported to have the highest burden of both Herpesviruses and HPV globally, thus presenting a unique region of interest.^11^^,^^12^ Furthermore, as the burden of orofacial tumors and oral cancers in particular gain worldwide prominence, there seems to be a corresponding rise in the disease in Ghana and SSA in general.^13^ Although several studies have been done in the sub-region to describe the epidemiology of orofacial tumors and oral cancers, much less has been done to explore the relationship between risk factors and oral cancers for this distinctive environment, particularly with respect to viruses.^12^ Considering the high prevalence of Herpesviruses and HPV in SSA, as well as the reported associations between these viruses and orofacial tumors, it is important that these associations are investigated for the uniquely burdened SSA region. This study therefore sought to determine the association of HPV and Herpesviruses in saliva and tumor tissue samples of patients with benign and malignant orofacial tumors, thereby exploring their use as possible biomarkers in the management of orofacial tumors.

## Materials and methods

### Study design and location

This was a cross-sectional, comparative clinical study of patients with benign and malignant oro-facial tumors reporting to the Department of Oral and Maxillofacial Surgery (OMFS), of the Korle-Bu Teaching Hospital (KBTH), Accra, and laboratory analysis done at the Noguchi Memorial Institute of Medical Research (NMIMR), and Virology Unit, Department of Medical Microbiology of the School of Medicine, University of Ghana.

### Study population

The study population consisted of patients who presented to the study site on account of benign and malignant orofacial tumors. Saliva samples were taken for a comparative group of asymptomatic patients at the same study site. The criteria for selecting eligible subjects for this study were patients presenting to the OMFS Department with an orofacial tumor confirmed subsequently by histology, patients who were 18 years and above, and patients who consented to be part of the study. Exclusion criteria for the study were patients who were severely ill or weak to have a biopsy taken, and patients with co-morbid conditions in whom biopsies were contra-indicated, such as severe renal or hepatic impairment, and patients with coagulopathies. Other criteria for exclusion were patients who were on anticoagulant therapy, patients who declined to have a biopsy of lesion done, patients who declined to have blood samples taken for laboratory investigations, patients who declined to give saliva samples for microbiological investigations, patients who were allergic to any of the materials used in the study, and referred patients who had received failed treatment from elsewhere.

### Sampling

Patients were recruited consecutively based on the inclusion criteria for the study. Clinical examination, and preliminary investigations were done for all patients, and initial management was carried out in accordance with the standard operating procedures and treatment guidelines of KBTH. Once the patient was stable, and a clinical impression of a benign or malignant orofacial tumor was made, the patient was then recruited to be part of the study.

### Variables and data collection

Independent variables for the study included the following: age, sex and employment status of the participants. Other independent variables were the level of education, employment (formal/informal/unemployed), National Health Insurance Scheme (NHIS) status (beneficiary/non-beneficiary), residence (Rural/Urban), duration of symptoms, anatomical location, comorbidities, tumor staging, family history, and presence of risk factor. The presence of risk factors was indicated as present if the patient smoked, had ever used tobacco, or frequently consumed alcohol (more than 600 mls a day, or 14 drinks a week). The outcome variables for the study included the following: HPV types in saliva, HPV types in tissue samples, Herpesvirus in saliva, Herpesvirus in tissue samples, and Hospital Length of Stay (LOS). The LOS was defined as the duration (in days), from admission to discharge (or death) for the selected treatment modality for each patient. Admission for patients was done a day prior to surgeries.

A structured questionnaire and data extraction sheet were used for data collection. This was made up of closed, and open-ended questions which were obtained directly from an interview, supplemented with the medical records of the patients.

Patients with orofacial tumors presented to the study site as walk-in patients, in emergency states, or patients referred from other facilities. Thorough history, clinical examinations and preliminary investigations were done for all patients with orofacial tumors. Depending on the degree of general illness of the patient with orofacial tumors, they may have required resuscitation and some initial management to alleviate pain and discomfort. Once the patient was stable, and met the inclusion criteria, they were spoken to concerning the study, and informed consent obtained.

**Fig. 1:**
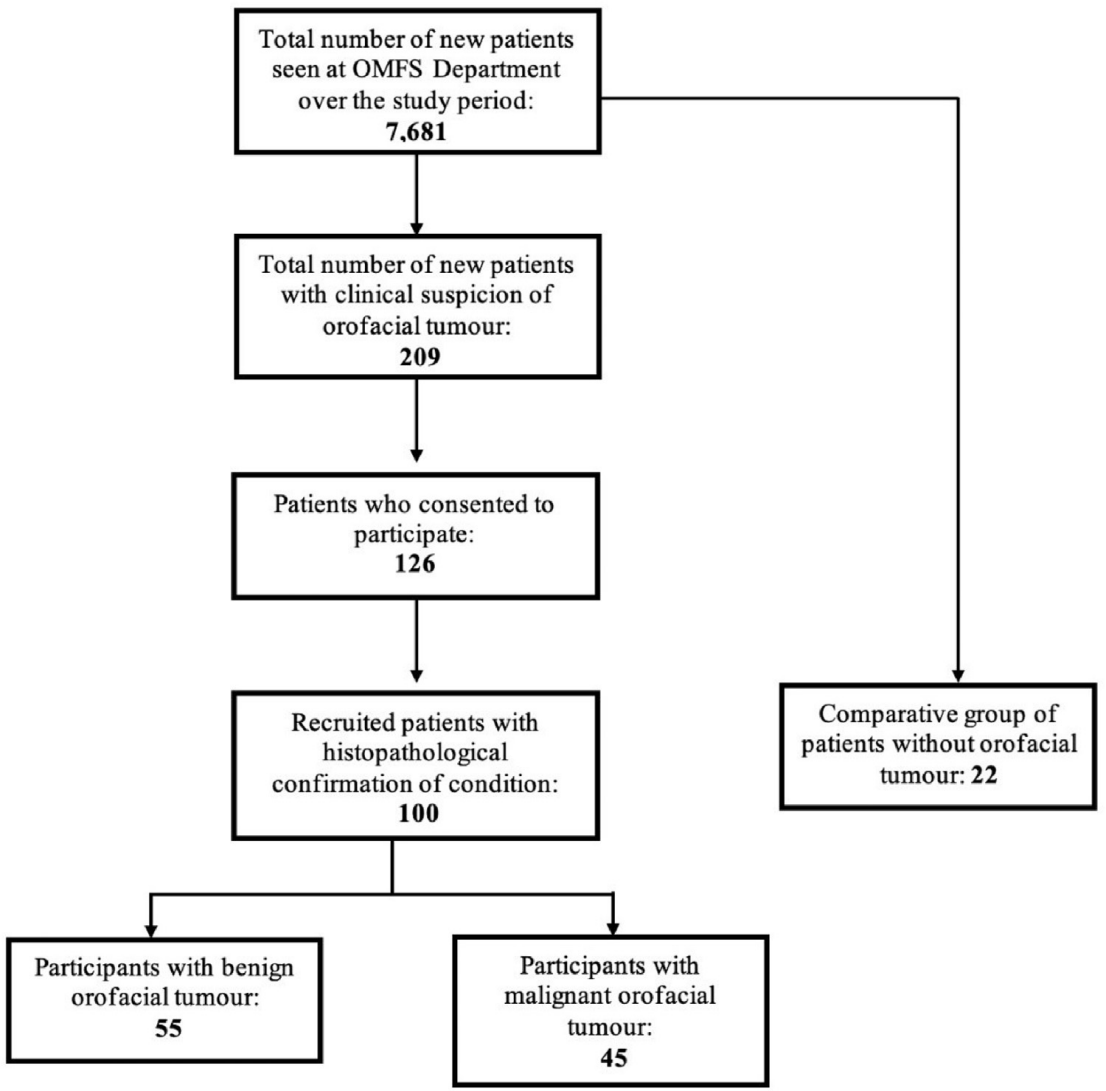
Recruitment summary of study participants.

Saliva collection was a non-invasive procedure that was done for all participants of the study, including the comparative group, consisting of individuals without orofacial tumors (Fig. 1). For the patients with orofacial tumors, this was done before the biopsy procedure. Participants were asked to wait for 30 minutes after eating, drinking, smoking, or chewing gum, before collection of the saliva sample. Unstimulated saliva was obtained with the patient sitting, as unstimulated saliva is superior in diagnosing biomarkers.^14^ Saliva was allowed to accumulate on the floor of the mouth, and the patient asked to spit into an open cryovial. It was ensured that the type of saliva obtained was without bubbles. The lid was closed tightly and transferred immediately for storage at -80°C.

The biopsy procedure was likewise done for all patients with orofacial tumors that met the inclusion criteria. At an appropriate and convenient time, patients were seated comfortably in the dental chair. Regional block, local analgesia together with infiltrative techniques (where applicable) were used in anesthesia for orofacial tumors of both the maxilla and the mandible. Favorable oral tissue that accessed the tumor was immobilized far from the area and biopsied with non-toothed tweezers. A clean and defined incision was performed to obtain a slice of tissue. Soft tissue incisions were elliptical in shape, producing a “V” wedge that included both the lesion and clinically healthy margins. Where multiple lesions were present, multiple biopsies were taken. Haemostasis was secured, and patients monitored for about 30 minutes post-operatively before discharge on post-operative instructions and appropriate dose of Amoxicillin (Caps Amoxicillin 500 mg 8 hourly for 5 days), Metronidazole (Tabs Metronidazole 400 mg 12 hourly for 5 days) and an appropriate analgesic which was (Tabs Ibuprufen 400 mg 8 hourly for 5 days) and/or Paracetamol (Tabs Paracetamol 1g 6 hourly for 5 days). For extensive exophytic lesions that could not be sutured, biopsies were done at safe and strategic anatomical sites, and hemostasis was secured with firm pressure packing using gauze impregnated with Bismuth Iodoform Paraffin Paste (BIPP). Also, for deeper-lying lesions associated with vital structures, biopsies were obtained with the use of a true-cut biopsy needle. All biopsy procedures were performed by the same investigator.

All participants, depending on the outcome of their histopathology reports were further managed according to the standard guidelines of the Oral and Maxillofacial Surgery unit. Treatment protocols included surgery and/or radiotherapy, palliative care, conservative management, and rehabilitative/supportive care. Patients were further monitored and followed up during their period of management.

### Molecular microbiology procedure

DNA was extracted from both the salivary and biopsy samples using the Quick-DNA Miniprep Kit (Zymo Research, USA), following the manufacturer's protocol. The nested-multiplex PCR method was utilized to identify 18 broad-spectrum high and low-risk HPV genotypes (6/11, 16, 18, 31, 33, 35, 39, 42, 43, 44, 45, 51, 52, 56, 58, 59, 66 and 68).^15^ Primers were selected based on the E6/E7 oncogenes, using outer consensus primers GP-E6F as the forward primer and 2 reverse primers GP-E7-5B and GP-E7-6B. Type-specific inner primers were combined in a cocktail to identify the specific genotypes, and the procedure was carried out as described before.^15^ Primers and combinations for the HPV cocktail were listed in other studies.^16^ For the Herpesviruses (HSV-1, HSV-2, Varicella-zoster virus, EBV, human cytomegalovirus, human herpesvirus 6, human herpesvirus 7, and Kaposi's sarcoma-associated herpesvirus) multiplex PCR was used. The procedure has previously been described.^17^ (The primers used^15^, ^17^ are listed in provided Supplementary Material).

The amplicons were visualized via gel electrophoresis. Results from analyses from the microbiological analyses were then recorded into a computerized questionnaire and specifically identified organisms and strain(s) recorded for each patient.

### Data analyses

All completed data were validated and entered into a Microsoft Excel (2010) entry sheet after each patient. Descriptive summaries were reported for the collected independent variables. Descriptive summaries were used to represent the HPV/HV present, in the saliva and tissue samples of the participants. A Chi-Square test was done to explore the distribution of the presence of HPV/HV in patients with orofacial tumors and non-tumorous comparative groups. Cross-tabulation was also done to compare the presence of HPV/HV among the background characteristics. Binary logistic regression was consequently used to determine any possible association. These were done at a 95 % confidence level, assuming an alpha level of 0.05.

### Quality control and ethical issues

Ethical approval was obtained from the Institutional Review Board of the Korle-Bu Teaching Hospital, Accra, Ghana (KBTH/STC/IRB/000136/2019), ensuring the ethical standards of the Institutional Research Committee and the Helsinki Declaration. The manuscript was also produced in conformity to the STROBE guidelines.

## Results

### Orofacial tumor characteristics

There was a total of 100 patients included in the study, consisting of 45 females (45 %) and 55 males (55 %). Ranging from 18 to 88 years, the mean age was 42.5 ± 19.5 years. Of the 100 study participants, 55 were histologically confirmed to have benign orofacial tumors and 45 malignant lesions. Most benign tumors were ameloblastoma (52.7 %), and fibro-osseous lesions (27.3 %). Odontogenic myxoma and Adenomatoid odontogenic tumours accounted for 5.4 % and 3.6 % of benign tumors respectively. On the other hand, seventy-one percent of malignant orofacial tumors were found to be Squamous Cell Carcinoma (SCC).

### Herpesviruses in biopsy samples and saliva of patients with orofacial tumors

Multiplex PCR was successfully completed for 91 biopsy tissue samples, 87 saliva samples and 22 saliva samples of asymptomatic controls. In all, the prevalence of herpesvirus in orofacial tumors was found to be 17.6 %, and their prevalence in the saliva of patients with orofacial tumors was 48.3 %. Overall, the herpesviruses detected in either tumor or saliva samples of patients with orofacial tumors were EBV (21.3 %), HHV-7 (11.2 %), CMV (6.7 %), HSV-1 (5.1 %), HSV-2 (1.1 %), VZV (1.1 %), and Kaposi sarcoma virus (0.6 %).

Herpes-simplex virus-1 and VZV were not detected in biopsy samples but were found in the saliva of patients with orofacial tumors. Human herpesvirus-6 was however neither detected in tumor samples nor saliva of patients (Table 1). Kaposi sarcoma virus was found in one tumor sample which was consistent with the patient's histological diagnosis of Kaposi's sarcoma. In saliva, HHV-7 was six times more likely in patients with benign tumors, compared to patients with malignant tumors.

**Table 1:** Prevalence of herpesviruses in saliva and tumor samples.

	Biopsy			Saliva		
Herpesvirus types	Benign tumors (∑ = 51)	Malignant tumors (∑ = 40)	X^2^ (p-value)	Benign tumors (∑ = 47)	Malignant tumors (∑ = 40)	bX^2^ (p-value)
HSV-1	0	0	‒	6 (12.8 %)	3 (7.5 %)	0.498
HSV-2	0	0	‒	1 (2.1 %)	1 (2.5 %)	0.647
Cytomegalovirus	5 (9.8 %)	0	0.065	5 (10.6 %)	2 (5.0 %)	0.445
Epstein-Barr virus	5 (9.8 %)	3 (7.5 %)	0.7	12 (25.5 %)	18 (45.0 %)	0.072
Varicella-zoster virus	0	0	‒	1 (2.1 %)	1 (2.5 %)	0.647
HHV-6	0	0	‒	0	0	‒
HHV-7	5 (9.8 %)	1 (2.5 %)	0.224	12 (25.5 %)	2 (5.0 %)	0.017^a^
Kaposi virus	0	1 (2.5 %)	0.44	0	0	‒
All herpesviruses	12 (23.5 %)	4 (10.0 %)	0.103	23 (48.9 %)	19 (47.5 %)	0.968
Co-infection	3 (5.9 %)	1 (2.5 %)		14 (29.8 %)	8 (20 %)	

a Fishers Exact test applied where applicable.

b Statistically significant.

EBV was found to be present in more patients with malignant orofacial tumors. The odds of EBV being detected in a patient with a malignant orofacial tumor was 2 times that of benign orofacial tumors (p = 0.072).

The prevalence of herpesvirus in the healthy control population was 22.7 % with multiple virus detection in two patients (Table 2). The odds of detecting herpesvirus in patients with orofacial tumors was 3.2 times that of patients without orofacial tumors. This association was found to be statistically significant (p-value = 0.032). There were four cases (4.4 %) in the study population in whom herpesvirus detection in tumors corresponded with what was found in saliva. Three of these were EBV innmalignant orofacial tumors. A logistic regression analysis done to explore the relationship between the detection of a virus in both tumor and saliva of the same patient, among study variables (sex, age category, tumor category, presence of risk factors), yielded no observable significant association.

**Table 2:** Prevalence of herpesviruses in saliva of patients with orofacial tumors compared with controls.

	Saliva of orofacial tumors		Saliva of controls		
Herpesviruses	Number	Percent	Number	Percent	aX^2^ (p-value)
HSV-1	8	9.2	0	0.0	0.683
HSV-2	2	2.3	1	4.5	0.600
Cytomegalovirus	7	8.0	1	4.5	0.574
Epstein-Barr virus	30	34.5	3	13.6	0.360
Varicella-zoster virus	2	2.3	0	0.0	1.000
HHV-6	0	0.0	0	0.0	‒
HHV-7	14	16.1	2	9.1	0.518
Kaposi virus	0	0.0	0	0.0	‒
All herpesviruses	63	48.3	7	17.6	0.033^b^

a Fishers Exact test applied where applicable.

b Statistically significant.

### HPV in tumor samples and the saliva of patients with orofacial tumors

In order to identify HPV, nested multiplex PCR was conducted for 90 biopsy tissues, 86 saliva samples, and 22 saliva samples of the asymptomatic control population. Overall, the genotypes detected in either tumor or saliva samples of patients with orofacial tumors were HPV-42 (29 %), HPV-43 (22.7 %), HPV-52 (22.2 %), HPV-39 (18.8 %), HPV-18 (9.1 %), HPV-35 (9.1 %), HPV-59 (8.5 %), HPV-31 (8.0 %), HPV-16 (4.5 %), and HPV-68 (4.5 %). HPV 66, 58, 45, 33, 56, 51, and 6 or 11 together were detected in 16.5 % of either biopsy or saliva samples of patients with orofacial tumors (Table 3).

**Table 3:** Prevalence of HPV in saliva and tumor samples of patients with orofacial tumors.

	Biopsy tissue			Saliva samples		
HPV Genotypes	Benign tumors (∑ = 50)	Malignant tumors (∑ = 40)	X^2^ (p-value)	Benign tumors (∑ = 46)	Malignant tumors (∑ = 40)	aX^2^ (p-value)
16	2 (4 %)	2 (5 %)	0.426	2 (4.4 %)	2 (5.0 %)	0.886
18	4 (8 %)	5 (12.5 %)	0.504	5 (10.9 %)	2 (5.0 %)	0.442
31	3 (6 %)	2 (5 %)	0.837	4 (8.7 %)	5 (12.5 %)	0.728
59	3 (6 %)	2 (5 %)	0.837	7 (15.2 %)	3 (7.5 %)	0.327
45	0	0	-	3 (6.5 %)	2 (5.0 %)	0.764
33	1 (2 %)	1 (2.5 %)	1.000	1 (2.2 %)	0	0.764
6 or 11	1 (2 %)	0	0.368	1 (2.2 %)	0	0.348
58	2 (4 %)	3 (7.5 %)	0.652	0	1 (2.5 %)	0.465
52	11 (22 %)	10 (25 %)	0.805	8 (17.4 %)	10 (25.0 %)	0.434
56	0	1 (2.5 %)	0.444	1 (2.2 %)	1 (2.5 %)	0.920
35	4 (8 %)	4 (10 %)	0.740	4 (8.7 %)	4 (10.0 %)	0.835
42	18 (36 %)	19 (47.5 %)	0.290	7 (15.2 %)	7 (17.5 %)	0.779
43	4 (8 %)	5 (12.5 %)	0.504	16 (34.8 %)	15 (37.5 %)	0.825
44	0	0	‒	0	0	‒
All HPV	30 (60 %)	27 (67.5 %)	0.514	30 (65.2 %)	30 (75.0 %)	0.342
Co-infection	15 (30 %)	14 (35 %)	0.293	19 (41.3 %)	14 (35.0 %)	0.381

a Fishers Exact test applied where applicable.

Prevalence of HPV DNA was higher in saliva samples compared to biopsy tissues (60.0 % vs. 57.0 %). The prevalence of HPV DNA in the saliva of the asymptomatic control population was however 18.2 % (four study participants). Among the benign tumor samples, the prevalence of HPV DNA was 60 %, compared to 67.5 % of the malignant tumor samples. Among the saliva of patients with benign tumors, however, the prevalence of HPV DNA was 65.2 %, compared to 75 % of the saliva of patients with malignant orofacial tumors (Table 3). The most prevalent genotypes in malignant tumors were HPV 42, 52, and 18 (48 %, 25 %, and 12.5 % respectively), while the most prevalent genotypes in the saliva of patients with malignant tumors were HPV 43, 52 and 42 (38 %, 25 %, and 18 % respectively). For both HPV 16 and 18, there was no observable difference in their presence between benign and malignant orofacial tumors for both tissue and saliva samples.

There was evidence of coinfection (presence of more than one genotype detected) in 29 tumor samples, which accounted for 35 % of malignant tumors, and 30 % of benign tumors. In saliva samples, however, more coinfections were detected in the saliva of patients with benign tumors (Table 4).

**Table 4:** Prevalence of HPV in the saliva of patients with orofacial tumors compared with controls.

	Saliva of patients with odontogenic tumors (86)		Control (22)		
HPV Genotypes	Number	Percent (%)	Number	Percent (%)	aChi-square (p-value)
16	4	4.7	0	0.0	0.580
18	7	8.1	0	0.0	0.341
31	9	10.5	0	0.0	0.199
59	10	11.6	0	0.0	0.093
45	5	5.8	0	0.0	0.581
33	1	1.2	0	0.0	0.611
6 or 11	1	1.2	0	0.0	0.611
58	1	1.2	0	0.0	0.611
52	18	20.9	4	18.2	0.775
56	2	2.3	0	0.0	0.470
35	8	9.3	0	0.0	0.203
42	14	16.3	2	9.1	0.517
43	31	36.1	2	9.1	0.018
44	0	0.0	0	0.0	‒
All HPV	60	69.7	3	18.2	<0.001^b^
Co-infection	33	38.4	2	13.6	0.023^b^

a Fishers Exact test applied where applicable.

b Statistically significant.

The most prevalent HPV genotypes in the saliva of all patients with orofacial tumors were HPV 43, 52, and 42 (36 %, 21 % and 16 %). The prevalence of HPV DNA in the saliva of patients with orofacial tumors was 69.7 %, compared to 18.2 % of the control sample. This difference was found to be statistically significant (p < 0.001).

HPV coinfection existed in 13.6 % of the control sub-population, compared to 38.4 % of patients with orofacial tumors (p = 0.023). There were thirteen cases (16 %) in the study population in whom the specific HPV detected in tumors corresponded with what was found in saliva. Six of these were malignant orofacial tumors, and seven were benign. A logistic regression done to explore the relationship between the detection of a virus in both tumor and saliva of the same patient, among study variables (sex, age category, tumor category, presence of risk factors), yielded no observable significant association. The odds of detecting herpesvirus in the poorly educated participants (the highest educational level being primary) was 1.33 times that of well-educated participants. Similarly, those with a positive history of a risk factor were 53 % more likely to have a herpesvirus detected. However, none of these associations were statistically significant. Also, participants with malignant tumors were more likely to have HPV detection (OR = 1.67). Similarly, those with a positive history of a risk factor were 7 % more likely to have HPV detected. Again, among patients with orofacial tumors, females were 1.88 times more likely to have HPV detected in either tumor or saliva. These associations, however, did not yield statistical significance. In all, squamous cell carcinoma was the malignant orofacial tumor with the highest prevalence of detectable viruses in saliva.

The overall hospital stay for study participants was 8.5 ± 6.7 days, with a median of 6.5 days (IQR: 3.5, 11.5). Those in whom viruses were detected spent a median of 7 days on admission compared to 5 days for participants who did not have any virus detected. Patients who had both viruses (herpesviruses and HPV) detected stayed at the hospital for a median of 14 days, compared to only herpesviruses or HPV (5 vs. 7 days). The differences in median lengths of stays were however not statistically significant.

## Discussion

This current study set out to explore the association of HPV and HV in saliva and tumor tissue samples of patients with orofacial tumors. Herpesvirus and HPV DNA were significantly more in the saliva of patients with orofacial tumors than in the asymptomatic control population. There was a considerably high prevalence of viruses in patients with orofacial tumors.

Herpesviruses, particularly HSV-1 and HSV-2 are known to be prevalent in the global population. Characterized by lifelong infections, the prevalence of HSV-1 has been reported to be as high as 88 % in some African populations.^18^ While HSV-1 and 2 have not been widely known to be oncogenic, it has been suggested that they could play a role in carcinogenesis by cellular protein induction, targeted chromosomal changes, and even stimulation of other viruses.^19^ This study did not show any difference in HSV 1 or 2 between benign and malignant tumors, or between the saliva of patients with orofacial tumors and controls. Similarly, Jain found no difference between HSV-1 IgG levels in cancerous and precancerous lesions.^20^

The mechanism of EBV is reported to be from interactions between EBV latent genes and oncogenes leading to host cell cycle disturbances, including the promotion of G1/S phase transition and inhibition of cell apoptosis, thereby fostering the development of neoplasms.^21^^,^^22^ There are several associations in literature between the EBV and carcinogenesis, particularly OSCC, lymphoma, nasopharyngeal carcinoma, and gastric cancer.^21^ In corroboration with this, the odds of EBV being detected in a patient with a malignant orofacial tumor was 2 times that of benign orofacial tumors in this study.

Again, there was an observable difference in the overall herpesvirus detection between the saliva of all patients with orofacial tumors, and the saliva of the control group in this study. While this observation might be consistent with the suggestion that herpesvirus co-infection might play a role in the development of solid tumors,^19^ an important potential interacting factor could be the presence of periodontal disease. The periodontal state of this study's participants was not determined. It is however evident from the literature that it could be a possible confounder in establishing the association between herpesviruses and orofacial tumors, especially when using biomarkers from saliva. Several studies have highlighted a relationship between various herpesviruses and periodontitis^23^^,^^24^ though the direction of the association is unclear. This could imply that the significant difference observed in the detection of herpesviruses between patients with orofacial tumors and healthy controls in this study could rather have been because the patients’ conditions did not allow for proper oral hygiene, thereby leading to periodontal disease, with possible consequent viral replication.

Oncogenic human papillomaviruses have for some time, been reported to be important aetiopathologic factors for Head and Neck Cancers (HNCs), with the classification of 16, 18, 31, 33, 35, 45, 52, 56, 58, and 59 as high-risk, and 6/11, 42, 43, and 44 as low-risk.^15^ In this study, apart from HPV-44, all the investigated genotypes were seen in either tumor or saliva of patients with orofacial tumors. The proportion of participants with detectable HPV was significant, compared to the control sample. Several other studies did not obtain high prevalence of HPV in HNCs: Blumberg et al., found none of the OSCC and oropharyngeal cancer tissues of Mozambican patients to stain positive for HPV.^25^ Kofi et al. also found an overall HPV prevalence of 0.74 % in a Central African population,^26^ likewise the 3.4 % and 23 % in Ghanaian populations by Dawson et al. and Aboagye et al. respectively.^16^^,^^27^ Compared to Dawson et al. and Aboagye et al., this study's overall higher prevalence in tissue biopsies could be accounted for by the fact that detection in the previous publications was done on formalin-fixed, paraffin-embedded specimens samples, and analyzed for very few genotypes. Archived samples are known to yield ultralow quantities of DNA.^28^

The prevalence of HPV in oropharyngeal cancers was estimated by Kim to be up to 60 %‒80 %, and demonstrable in just about 20 %, of oral cancers.^26^ Other reviews however suggest a much higher prevalence for the presence of HPV, up to over 90 %, especially for HPV-16.^27^ Though the overall HPV prevalence in this study was a comparable 67.5 % in malignant tumors, the prevalence of HPV 16 and 18, in particular, were 5 % and 12.5 % respectively. The most prevalent genotype in this current study was HPV-42 (in tissue) and HPV-43 in saliva samples. With respect to the prevalence of HPV in the asymptomatic population, it has been reported to be 5 %‒19.6 % in American young populations,^27^ 5.6 % in South African men,^29^ and 35 % in the Australian population.^30^ The burden of oral HPV in the asymptomatic population favorably compares to the 18.2 % found in this study.

This study suggests a substantial difference in the prevalence of HPV in orofacial tumors in general, compared to the control population. There was no indication of a considerable difference in HPV burden between benign and malignant tumors. This observation might support the assertion that like SCC, HPV could also have a role to play in benign oral tumors as well. In an Iranian systematic review, Mravak-Stipetić et al. found HPV in a considerable amount of benign lesions (19 %), as well as 7 % of normal mucosa.^31^ Specifically for ameloblastoma which constituted the majority of benign orofacial tumors, Sand et al. found HPV in 67 % of ameloblastoma samples tested, likewise in several other studies.^32^ The role of viruses in benign orofacial tumors, if any, is unclear from the literature, but questions have arisen to probe if these viruses enter the tissues from the oral cavity, or if the tumor tissues get contaminated by oral fluids in the process of taking the biopsy. Similar to herpesviruses, there is also evidence to suggest that HPV might be related to a characteristic periodontal microbiome,^33^ which could imply that its high prevalence in patients with orofacial tumors could in part be due to their poor oral hygiene practices.

Limitations of this study include the observation that its small sample size hindered the sub-group analyses. Furthermore, the sampling of patients from only one facility could also affect the external validity of the study's results. Findings from this study however provide invaluable information regarding the potential role of HPV and Herpesviruses in orofacial tumors. Another strength of this study is the presentation of a very elaborate method that can be adopted by clinicians for further exploration in this field.

In conclusion, this study found a high prevalence of Herpesviruses and HPV in saliva and tumor samples of patients with orofacial tumors, with EBV being more likely to be found in malignant tumor samples. Viruses were more likely to be detected in the saliva of patients with orofacial tumors than the asymptomatic control population.

## Ethical approval

Ethical approval was obtained from the Institutional Review Board of the Korle-Bu Teaching Hospital, Accra, Ghana (KBTH/STC/IRB/000136/2019), ensuring the ethical standards of the Institutional Research Committee and the Helsinki Declaration.

## Funding

This research did not receive any specific grant from funding agencies in the public, commercial, or not-for-profit sectors.

## Declaration of competing interest

The authors declare that they have no known competing financial interests or personal relationships that could have appeared to influence the work reported in this paper.

## Appendix Supplementary materials

Image, application 1
