# Supplementary material for: Herpesviruses and human papillomaviruses in saliva and biopsies of patients with orofacial tumors
Source: Clinics (Sao Paulo). 2024 Aug 31;79:100477. doi: 10.1016/j.clinsp.2024.100477 (PMC11402418; doi:10.1016/j.clinsp.2024.100477)
Supplement: Supplementary file 1 [file mmc1.docx]

CLINICS-D-23-00714_ **Supplementary Material**

**Supplementary Material**

**Supplementary Table 1** Primers of herpesviruses detected in saliva and biopsies of patients with orofacial tumors.

| **Virus** | **Specific Gene/Protein** | **Primer sequences** |
| --- | --- | --- |
| HSV1 | UL30 | TGTTTCGCGTGTGGGACATA |
|  |  | GCAAAGTCGAACACCACCAC |
| HSV2 | UL30 | TCAGCGAGGATAACCTGGGA |
|  |  | AAAACGCAATACCGCCGATG |
| HHV6 | IE1 protein (U90) gene | CAAGCCCTAACTGTGTATGT |
|  |  | TCTGCAATGTAATCAGTTTC |
| HCMV | Major capsid protein gene | GAGCGCGTCCACAAAGTCTA |
|  |  | GTGATCCGACTGGGCGAAAA |
| VZV | ORF8/ORF9 gene | CACACGATAATGCCTGATCGG |
|  |  | TGCTGATATTTCCACGGTACAGC |
| EBV | Highly conserved region within DNA polymerase gene | AACATTGGCAGCAGGTAAGC |
|  |  | ACTTACCAAGTGTCCATAGGAGC |
| HHV-7 | U84 gene | CAGACATCGATACAGAGTTTGA |
|  |  | ATTTCCGGAATGTAGCCAACAA |
| HHV-8 | ORF26 gene (putative minor capsid protein) | AGCCGAAAGGATTCCACCAT |
|  |  | TCCGTGTTGTCTACGTCCAG |

Primers of human papilloma viruses detected in saliva and biopsies of patients with orofacial tumors.

| **Primer cocktail** | **HPV genotype** | **Amplicon (bp)** | **Sequence (5'-3')** | **Position (bp)** |
| --- | --- | --- | --- | --- |
| I | 16 | 457 | CAC AGT TAT GCA CAG AGC TGC | 141–161 |
|  |  |  | CAT ATA TTC ATG CAA TGT AGG TGT A | 597–573 |
|  | 18 | 322 | CAC TTC ACT GCA AGA CAT AGA | 170–190 |
|  |  |  | GTT GTG AAA TCG TCG TTT TTC A | 491–470 |
|  | 31 | 263 | GAA ATT GCA TGA ACT AAG CTC G | 137–158 |
|  |  |  | CAC ATA TAC CTT TGT TTG TCA A | 399–378 |
|  | 59 | 215 | CAA AGG GGA ACT GCA AGA AAG | 159–179 |
|  |  |  | TAT AAC AGC GTA TCA GCA GC | 373–354 |
|  | 45 | 151 | GTG GAA AAG TGC ATT ACA GG | 82–101 |
|  |  |  | ACC TCT GTG CGT TCC AAT GT | 232–213 |
| II | 33 | 398 | ACT ATA CAC AAC ATT GAA CTA | 172–192 |
|  |  |  | GTT TTT ACA CGT CAC AGT GCA | 569–549 |
|  | 6/11 | 334 | TGC AAG AAT GCA CTG ACC AC | 201–220 |
|  |  |  | TGC ATG TTG TCC AGC AGT GT | 534–515 |
|  | 58 | 274 | GTA AAG TGT GCT TAC GAT TGC | 297–317 |
|  |  |  | GTT GTT ACA GGT TAC ACT TGT | 570–550 |
|  | 52 | 229 | TAA GGC TGC AGT GTG TGC AG | 178–197 |
|  |  |  | CTA ATA GTT ATT TCA CTT AAT GGT | 406–383 |
|  | 56 | 181 | GTG TGC AGA GTA TGT TTA TTG | 294–314 |
|  |  |  | TTT CTG TCA CAA TGC AAT TGC | 475–455 |
| III | 35 | 358 | CAA CGA GGT AGA AGA AAG CAT C | 157–178 |
|  |  |  | CCG ACC TGT CCA CCG TCC ACC G | 514–493 |
|  | 42 | 277 | CCC AAA GTA GTG GTC CCA GTT A | 85–106 |
|  |  |  | GAT CTT TCG TAG TGT CGC AGT G | 361–340 |
|  | 43 | 219 | GCA TAA TGT CTG CAC GTA GCT G | 102–123 |
|  |  |  | CAT GAA ACT GTA GAC AGG CCA AG | 320–298 |
|  | 44 | 163 | TAA ACA GTT ATA TGT AGT GTA CCG | 248–271 |
|  |  |  | TAT CAG CAC GTC CAG AAT TGA C | 410–389 |

**Supplementary Table 2** DNA concentrations of collected samples (factor 50).

| **Sample ID** | **User name** | **Nucleic Acid** | **Unit** | **A260 (Abs)** | **A280 (Abs)** | **260/280** | **260/230** |
| --- | --- | --- | --- | --- | --- | --- | --- |
| BLANK | Geldoc PC | -0.1 | ng/µL | -0.002 | -0.006 | 0.27 | 0.07 |
| B001 | Geldoc PC | 49.3 | ng/µL | 0.986 | 0.536 | 1.84 | 0.32 |
| B002 | Geldoc PC | 60.4 | ng/µL | 1.208 | 0.658 | 1.84 | 0.23 |
| B003 | Geldoc PC | 80.2 | ng/µL | 1.605 | 0.878 | 1.83 | 0.32 |
| B004 | Geldoc PC | 38.4 | ng/µL | 0.768 | 0.422 | 1.82 | 0.23 |
| B005 | Geldoc PC | 59.7 | ng/µL | 1.195 | 0.652 | 1.83 | 0.29 |
| B006 | Geldoc PC | 73.2 | ng/µL | 1.465 | 0.8 | 1.83 | 0.31 |
| B007 | Geldoc PC | 26 | ng/µL | 0.52 | 0.279 | 1.86 | 0.18 |
| B008 | Geldoc PC | 13.6 | ng/µL | 0.273 | 0.154 | 1.77 | 0.09 |
| B009 | Geldoc PC | 10.6 | ng/µL | 0.211 | 0.103 | 2.06 | 0.11 |
| B011 | Geldoc PC | 20.4 | ng/µL | 0.408 | 0.217 | 1.88 | 0.13 |
| B012 | Geldoc PC | 9.5 | ng/µL | 0.189 | 0.098 | 1.94 | 0.1 |
| B013 | Geldoc PC | 65.6 | ng/µL | 1.313 | 0.706 | 1.86 | 0.61 |
| B014 | Geldoc PC | 83.8 | ng/µL | 1.677 | 0.901 | 1.86 | 0.21 |
| B015 | Geldoc PC | 85.2 | ng/µL | 1.703 | 0.937 | 1.82 | 0.23 |
| B016 | Geldoc PC | 114.9 | ng/µL | 2.298 | 1.248 | 1.84 | 0.3 |
| B017 | Geldoc PC | 47.4 | ng/µL | 0.948 | 0.505 | 1.88 | 0.29 |
| B018 | Geldoc PC | 35.4 | ng/µL | 0.709 | 0.389 | 1.82 | 0.07 |
| B019 | Geldoc PC | 18.2 | ng/µL | 0.363 | 0.195 | 1.86 | 0.05 |
| B020 | Geldoc PC | 26.7 | ng/µL | 0.534 | 0.302 | 1.77 | 0.08 |
| B021 | Geldoc PC | 21.1 | ng/µL | 0.422 | 0.218 | 1.93 | 0.08 |
| B023 | Geldoc PC | 6.9 | ng/µL | 0.137 | 0.067 | 2.04 | 0.06 |
| B024 | Geldoc PC | 64.2 | ng/µL | 1.284 | 0.697 | 1.84 | 0.56 |
| B025 | Geldoc PC | 87 | ng/µL | 1.741 | 0.927 | 1.88 | 1.16 |
| B026 | Geldoc PC | 40.4 | ng/µL | 0.808 | 0.431 | 1.88 | 0.34 |
| B027 | Geldoc PC | 83.9 | ng/µL | 1.678 | 0.919 | 1.83 | 0.25 |
| B028 | Geldoc PC | 28.8 | ng/µL | 0.576 | 0.303 | 1.9 | 0.21 |
| B029 | Geldoc PC | 46.9 | ng/µL | 0.938 | 0.513 | 1.83 | 0.32 |
| B030 | Geldoc PC | 26.7 | ng/µL | 0.534 | 0.288 | 1.85 | 0.27 |
| B032 | Geldoc PC | 21.7 | ng/µL | 0.433 | 0.228 | 1.9 | 0.08 |
| B033 | Geldoc PC | 64.1 | ng/µL | 1.283 | 0.696 | 1.84 | 0.34 |
| B034 | Geldoc PC | 56 | ng/µL | 1.12 | 0.602 | 1.86 | 0.46 |
| B035 | Geldoc PC | 5.3 | ng/µL | 0.107 | 0.023 | 4.55 | 0.12 |
| B036 | Geldoc PC | 31.6 | ng/µL | 0.632 | 0.339 | 1.86 | 0.4 |
| B037 | Geldoc PC | 18.4 | ng/µL | 0.368 | 0.188 | 1.96 | 0.2 |
| B038 | Geldoc PC | 32.5 | ng/µL | 0.649 | 0.338 | 1.92 | 0.25 |
| B039 | Geldoc PC | 72.7 | ng/µL | 1.454 | 0.78 | 1.86 | 0.42 |
| B040 | Geldoc PC | 75.8 | ng/µL | 1.515 | 0.827 | 1.83 | 0.33 |
| B042 | Geldoc PC | 20.6 | ng/µL | 0.412 | 0.212 | 1.95 | 0.13 |
| B043 | Geldoc PC | 4.9 | ng/µL | 0.099 | 0.036 | 2.72 | 0.03 |
| B044 | Geldoc PC | 2.5 | ng/µL | 0.049 | 0.018 | 2.73 | 0.03 |
| B045 | Geldoc PC | 10.1 | ng/µL | 0.203 | 0.098 | 2.06 | 0.08 |
| B046 | Geldoc PC | 22.3 | ng/µL | 0.445 | 0.222 | 2.01 | 0.17 |
| B047 | Geldoc PC | 14.7 | ng/µL | 0.294 | 0.162 | 1.81 | 0.06 |
| B048 | Geldoc PC | 28.3 | ng/µL | 0.566 | 0.292 | 1.94 | 0.13 |
| B049 | Geldoc PC | 46.4 | ng/µL | 0.928 | 0.489 | 1.9 | 0.4 |
| B050 | Geldoc PC | 137.1 | ng/µL | 2.742 | 1.46 | 1.88 | 0.86 |
| B051 | Geldoc PC | 87.9 | ng/µL | 1.757 | 0.946 | 1.86 | 0.49 |
| B052 | Geldoc PC | 38 | ng/µL | 0.761 | 0.396 | 1.92 | 0.2 |
| B053 | Geldoc PC | 68.5 | ng/µL | 1.37 | 0.738 | 1.86 | 0.21 |
| B054 | Geldoc PC | 87.1 | ng/µL | 1.742 | 0.94 | 1.85 | 0.43 |
| B055 | Geldoc PC | 23.1 | ng/µL | 0.461 | 0.236 | 1.95 | 0.24 |
| B056 | Geldoc PC | 29.6 | ng/µL | 0.593 | 0.297 | 1.99 | 0.47 |
| B057 | Geldoc PC | 54.1 | ng/µL | 1.082 | 0.579 | 1.87 | 0.49 |
| B058 | Geldoc PC | 37.8 | ng/µL | 0.757 | 0.398 | 1.9 | 0.24 |
| B059 | Geldoc PC | 17.1 | ng/µL | 0.342 | 0.174 | 1.97 | 0.13 |
| B060 | Geldoc PC | 40 | ng/µL | 0.8 | 0.43 | 1.86 | 0.28 |
| B061 | Geldoc PC | 42.1 | ng/µL | 0.842 | 0.436 | 1.93 | 0.64 |
| B062 | Geldoc PC | 42.7 | ng/µL | 0.853 | 0.45 | 1.9 | 0.48 |
| B063 | Geldoc PC | 38.4 | ng/µL | 0.768 | 0.398 | 1.93 | 0.38 |
| B064 | Geldoc PC | 198.6 | ng/µL | 3.972 | 2.115 | 1.88 | 1.41 |
| B066 | Geldoc PC | 58.1 | ng/µL | 1.162 | 0.63 | 1.85 | 0.43 |
| B067 | Geldoc PC | 39 | ng/µL | 0.78 | 0.425 | 1.84 | 0.32 |
| B068 | Geldoc PC | 8.2 | ng/µL | 0.164 | 0.075 | 2.18 | 0.1 |
| B069 | Geldoc PC | 4.3 | ng/µL | 0.087 | 0.045 | 1.94 | 0.03 |
| B070 | Geldoc PC | 111 | ng/µL | 2.22 | 1.196 | 1.86 | 0.81 |
| B071 | Geldoc PC | 12.5 | ng/µL | 0.25 | 0.129 | 1.95 | 0.12 |
| B072 | Geldoc PC | 143.4 | ng/µL | 2.869 | 1.542 | 1.86 | 0.63 |
| B073 | Geldoc PC | 6.7 | ng/µL | 0.134 | 0.06 | 2.24 | 0.11 |
| B074 | Geldoc PC | 12.9 | ng/µL | 0.258 | 0.119 | 2.18 | 0.09 |
| B075 | Geldoc PC | 8.8 | ng/µL | 0.176 | 0.078 | 2.26 | 0.06 |
| B076 | Geldoc PC | 214.2 | ng/µL | 4.284 | 2.281 | 1.88 | 1.28 |
| B077 | Geldoc PC | 104.1 | ng/µL | 2.081 | 1.109 | 1.88 | 0.92 |
| B078 | Geldoc PC | 91.5 | ng/µL | 1.83 | 0.981 | 1.86 | 0.38 |
| B079 | Geldoc PC | 131.1 | ng/µL | 2.623 | 1.392 | 1.88 | 0.74 |
| B080 | Geldoc PC | 102.1 | ng/µL | 2.042 | 1.094 | 1.87 | 0.7 |
| B081 | Geldoc PC | 86.4 | ng/µL | 1.729 | 0.939 | 1.84 | 0.67 |
| B082 | Geldoc PC | 55.1 | ng/µL | 1.101 | 0.606 | 1.82 | 0.18 |
| B083 | Geldoc PC | 181.2 | ng/µL | 3.624 | 1.951 | 1.86 | 0.74 |
| B084 | Geldoc PC | 93.7 | ng/µL | 1.874 | 1.036 | 1.81 | 0.45 |
| B085 | Geldoc PC | 92.3 | ng/µL | 1.846 | 1.012 | 1.82 | 0.52 |
| B086 | Geldoc PC | 113.3 | ng/µL | 2.267 | 1.213 | 1.87 | 0.75 |
| B087 | Geldoc PC | 14.6 | ng/µL | 0.292 | 0.159 | 1.83 | 0.25 |
| B088 | Geldoc PC | 20.7 | ng/µL | 0.414 | 0.211 | 1.96 | 0.24 |
| B089 | Geldoc PC | 29.7 | ng/µL | 0.594 | 0.31 | 1.92 | 0.36 |
| B103 | Geldoc PC | 35.5 | ng/µL | 0.71 | 0.376 | 1.89 | 0.73 |
| B105 | Geldoc PC | 23.1 | ng/µL | 0.461 | 0.226 | 2.04 | 0.43 |
| B106 | Geldoc PC | 142.4 | ng/µL | 2.848 | 1.519 | 1.88 | 1.03 |
| B107 | Geldoc PC | 58.6 | ng/µL | 1.172 | 0.631 | 1.86 | 0.65 |
| B109 | Geldoc PC | 9.2 | ng/µL | 0.184 | 0.083 | 2.21 | 0.23 |
| B110 | Geldoc PC | 16 | ng/µL | 0.32 | 0.167 | 1.91 | 0.1 |
| B111 | Geldoc PC | 12.4 | ng/µL | 0.248 | 0.117 | 2.11 | 0.27 |
| B112 | Geldoc PC | 39.9 | ng/µL | 0.797 | 0.422 | 1.89 | 0.49 |
| B120 | Geldoc PC | 18.7 | ng/µL | 0.374 | 0.19 | 1.97 | 0.23 |
| B121 | Geldoc PC | 11.8 | ng/µL | 0.237 | 0.111 | 2.14 | 0.18 |
| B001C | Geldoc PC | 10.3 | ng/µL | 0.207 | 0.097 | 2.13 | 0.17 |
| B002C | Geldoc PC | 24.5 | ng/µL | 0.49 | 0.248 | 1.98 | 0.33 |
| B003C | Geldoc PC | 9.7 | ng/µL | 0.193 | 0.084 | 2.29 | 0.04 |
| B004C | Geldoc PC | 19.7 | ng/µL | 0.393 | 0.189 | 2.09 | 0.14 |
| B005C | Geldoc PC | 10.7 | ng/µL | 0.215 | 0.101 | 2.12 | 0.18 |
| B006C | Geldoc PC | 5 | ng/µL | 0.1 | 0.045 | 2.24 | 0.08 |
| B007C | Geldoc PC | 4.6 | ng/µL | 0.093 | 0.035 | 2.65 | 0.13 |
| B008C | Geldoc PC | 5.9 | ng/µL | 0.117 | 0.043 | 2.73 | 0.15 |
| B009C | Geldoc PC | 7.4 | ng/µL | 0.148 | 0.068 | 2.17 | 0.16 |
| B010C | Geldoc PC | 9 | ng/µL | 0.18 | 0.092 | 1.96 | 0.17 |
| B011C | Geldoc PC | 5.1 | ng/µL | 0.102 | 0.039 | 2.59 | 0.18 |
| B012C | Geldoc PC | 3.5 | ng/µL | 0.069 | 0.022 | 3.21 | 0.1 |
| B013C | Geldoc PC | 7.9 | ng/µL | 0.158 | 0.073 | 2.16 | 0.14 |
| B014C | Geldoc PC | 17.3 | ng/µL | 0.345 | 0.176 | 1.96 | 0.54 |
| B015C | Geldoc PC | 5.7 | ng/µL | 0.113 | 0.036 | 3.18 | 0.04 |
| S002 | Geldoc PC | 29.2 | ng/µL | 0.583 | 0.377 | 1.55 | 0.17 |
| S003 | Geldoc PC | 17.5 | ng/µL | 0.349 | 0.205 | 1.7 | 0.12 |
| S004 | Geldoc PC | 10.6 | ng/µL | 0.211 | 0.119 | 1.77 | 0.07 |
| S005 | Geldoc PC | 22.5 | ng/µL | 0.45 | 0.254 | 1.77 | 0.21 |
| S006 | Geldoc PC | 3.4 | ng/µL | 0.068 | 0.035 | 1.95 | 0.04 |
| S007 | Geldoc PC | 14.8 | ng/µL | 0.296 | 0.17 | 1.74 | 0.17 |
| S008 | Geldoc PC | 144.4 | ng/µL | 2.889 | 1.553 | 1.86 | 0.93 |
| S009 | Geldoc PC | 127.2 | ng/µL | 2.544 | 1.354 | 1.88 | 0.79 |
| S011 | Geldoc PC | 125.3 | ng/µL | 2.507 | 1.335 | 1.88 | 0.63 |
| S012 | Geldoc PC | 47.6 | ng/µL | 0.952 | 0.545 | 1.75 | 0.07 |
| S014 | Geldoc PC | 33.7 | ng/µL | 0.675 | 0.405 | 1.66 | 0.1 |
| S015 | Geldoc PC | 111.1 | ng/µL | 2.221 | 1.193 | 1.86 | 0.61 |
| S017 | Geldoc PC | 18 | ng/µL | 0.361 | 0.213 | 1.69 | 0.13 |
| S018 | Geldoc PC | 26.4 | ng/µL | 0.528 | 0.288 | 1.83 | 0.23 |
| S019 | Geldoc PC | 120.5 | ng/µL | 2.41 | 1.299 | 1.85 | 0.68 |
| S020 | Geldoc PC | 63.3 | ng/µL | 1.267 | 0.685 | 1.85 | 0.39 |
| S025 | Geldoc PC | 40.4 | ng/µL | 0.809 | 0.443 | 1.83 | 0.16 |
| S025 | Geldoc PC | 40.2 | ng/µL | 0.804 | 0.428 | 1.88 | 0.16 |
| S026 | Geldoc PC | 13 | ng/µL | 0.259 | 0.171 | 1.52 | 0.05 |
| S027 | Geldoc PC | 6.3 | ng/µL | 0.125 | 0.055 | 2.26 | 0.01 |
| S028 | Geldoc PC | 28.6 | ng/µL | 0.571 | 0.298 | 1.92 | 0.04 |
| S030 | Geldoc PC | 26.5 | ng/µL | 0.53 | 0.355 | 1.49 | 0.07 |
| S032 | Geldoc PC | 48.9 | ng/µL | 0.978 | 0.63 | 1.55 | 0.06 |
| S033 | Geldoc PC | 124.3 | ng/µL | 2.486 | 1.39 | 1.79 | 0.44 |
| S034 | Geldoc PC | 53 | ng/µL | 1.06 | 0.616 | 1.72 | 0.08 |
| S036 | Geldoc PC | 80.1 | ng/µL | 1.602 | 0.871 | 1.84 | 0.23 |
| S037 | Geldoc PC | 10.3 | ng/µL | 0.207 | 0.098 | 2.1 | 0.02 |
| S038 | Geldoc PC | 61.1 | ng/µL | 1.222 | 0.68 | 1.8 | 0.15 |
| S039 | Geldoc PC | 21.9 | ng/µL | 0.437 | 0.234 | 1.87 | 0.15 |
| S041 | Geldoc PC | 36 | ng/µL | 0.721 | 0.397 | 1.81 | 0.29 |
| S042 | Geldoc PC | 55.9 | ng/µL | 1.119 | 0.65 | 1.72 | 0.41 |
| S043 | Geldoc PC | 16.5 | ng/µL | 0.331 | 0.176 | 1.88 | 0.18 |
| S045 | Geldoc PC | 61.4 | ng/µL | 1.227 | 0.679 | 1.81 | 0.65 |
| S046 | Geldoc PC | 48.9 | ng/µL | 0.977 | 0.521 | 1.88 | 0.71 |
| S047 | Geldoc PC | 47.5 | ng/µL | 0.951 | 0.509 | 1.87 | 0.86 |
| S047 | Geldoc PC | 34.6 | ng/µL | 0.693 | 0.375 | 1.85 | 0.74 |
| S048 | Geldoc PC | 17 | ng/µL | 0.34 | 0.185 | 1.84 | 0.44 |
| S049 | Geldoc PC | 4.3 | ng/µL | 0.085 | 0.041 | 2.11 | 0.18 |
| S050 | Geldoc PC | 84.6 | ng/µL | 1.692 | 0.918 | 1.84 | 1.42 |
| S051 | Geldoc PC | 125 | ng/µL | 2.499 | 1.366 | 1.83 | 1.47 |
| S052 | Geldoc PC | 106.6 | ng/µL | 2.132 | 1.175 | 1.81 | 0.93 |
| S053 | Geldoc PC | 27.7 | ng/µL | 0.555 | 0.336 | 1.65 | 0.36 |
| S054 | Geldoc PC | 29.9 | ng/µL | 0.597 | 0.327 | 1.82 | 0.18 |
| S055 | Geldoc PC | 40.2 | ng/µL | 0.804 | 0.43 | 1.87 | 0.42 |
| S056 | Geldoc PC | 37.3 | ng/µL | 0.745 | 0.405 | 1.84 | 0.42 |
| S057 | Geldoc PC | 63.6 | ng/µL | 1.271 | 0.843 | 1.51 | 0.51 |
| S058 | Geldoc PC | 88 | ng/µL | 1.76 | 1.028 | 1.71 | 0.2 |
| S059 | Geldoc PC | 43.4 | ng/µL | 0.868 | 0.557 | 1.56 | 0.15 |
| S060 | Geldoc PC | 23.1 | ng/µL | 0.462 | 0.269 | 1.72 | 0.19 |
| S061 | Geldoc PC | 16.9 | ng/µL | 0.338 | 0.183 | 1.84 | 0.23 |
| S062 | Geldoc PC | 81.8 | ng/µL | 1.636 | 0.885 | 1.85 | 1.04 |
| S063 | Geldoc PC | 23.3 | ng/µL | 0.466 | 0.282 | 1.65 | 0.55 |
| S064 | Geldoc PC | 63.1 | ng/µL | 1.263 | 0.705 | 1.79 | 0.87 |
| S065 | Geldoc PC | 43.2 | ng/µL | 0.863 | 0.494 | 1.75 | 0.15 |
| S066 | Geldoc PC | 45.2 | ng/µL | 0.904 | 0.529 | 1.71 | 0.32 |
| S068 | Geldoc PC | 39.5 | ng/µL | 0.789 | 0.455 | 1.73 | 0.39 |
| S069 | Geldoc PC | 50.9 | ng/µL | 1.018 | 0.63 | 1.61 | 0.12 |
| S070 | Geldoc PC | 285.7 | ng/µL | 5.713 | 3.067 | 1.86 | 0.97 |
| S071 | Geldoc PC | 120.5 | ng/µL | 2.41 | 1.299 | 1.86 | 0.81 |
| S072 | Geldoc PC | 14 | ng/µL | 0.28 | 0.184 | 1.52 | 0.12 |
| S073 | Geldoc PC | 207.7 | ng/µL | 4.154 | 2.543 | 1.63 | 0.25 |
| S074 | Geldoc PC | 48.1 | ng/µL | 0.962 | 0.533 | 1.81 | 0.2 |
| S075 | Geldoc PC | 3.8 | ng/µL | 0.076 | 0.044 | 1.75 | 0.03 |
| S076 | Geldoc PC | 165.4 | ng/µL | 3.308 | 1.774 | 1.86 | 1.36 |
| S077 | Geldoc PC | 23.2 | ng/µL | 0.464 | 0.264 | 1.76 | 0.4 |
| S078 | Geldoc PC | 36.1 | ng/µL | 0.723 | 0.427 | 1.69 | 0.61 |
| S079 | Geldoc PC | 66.5 | ng/µL | 1.329 | 0.73 | 1.82 | 0.97 |
| S080 | Geldoc PC | 23.1 | ng/µL | 0.461 | 0.26 | 1.77 | 0.44 |
| S081 | Geldoc PC | 96 | ng/µL | 1.919 | 1.287 | 1.49 | 0.1 |
| S082 | Geldoc PC | 7.5 | ng/µL | 0.149 | 0.106 | 1.41 | 0.02 |
| S083 | Geldoc PC | 52 | ng/µL | 1.04 | 0.573 | 1.82 | 0.35 |
| S084 | Geldoc PC | 21.1 | ng/µL | 0.423 | 0.241 | 1.75 | 0.26 |
| S085 | Geldoc PC | 282.8 | ng/µL | 5.656 | 3.005 | 1.88 | 1.58 |
| S086 | Geldoc PC | 221.1 | ng/µL | 4.421 | 2.333 | 1.9 | 1.35 |
| S087 | Geldoc PC | 41.7 | ng/µL | 0.835 | 0.489 | 1.71 | 0.48 |
| S088 | Geldoc PC | 39.7 | ng/µL | 0.795 | 0.467 | 1.7 | 0.55 |
| S089 | Geldoc PC | 12.2 | ng/µL | 0.243 | 0.148 | 1.65 | 0.06 |
| S090 | Geldoc PC | 141.2 | ng/µL | 2.823 | 1.631 | 1.73 | 0.8 |
| S091 | Geldoc PC | 67.4 | ng/µL | 1.347 | 0.763 | 1.77 | 0.58 |
| S092 | Geldoc PC | 30.8 | ng/µL | 0.616 | 0.344 | 1.79 | 0.49 |
| S093 | Geldoc PC | 35.3 | ng/µL | 0.707 | 0.363 | 1.95 | 0.06 |
| S094 | Geldoc PC | 45.5 | ng/µL | 0.911 | 0.497 | 1.83 | 0.15 |
| S095 | Geldoc PC | 78.7 | ng/µL | 1.574 | 0.884 | 1.78 | 0.37 |
| S096 | Geldoc PC | 148 | ng/µL | 2.959 | 1.57 | 1.88 | 0.24 |
| S097 | Geldoc PC | 251.2 | ng/µL | 5.024 | 2.676 | 1.88 | 0.79 |
| S098 | Geldoc PC | 110.6 | ng/µL | 2.213 | 1.233 | 1.79 | 0.2 |
| S099 | Geldoc PC | 88.1 | ng/µL | 1.762 | 0.956 | 1.84 | 0.26 |
| S100 | Geldoc PC | 44 | ng/µL | 0.88 | 0.492 | 1.79 | 0.22 |
| S101 | Geldoc PC | 466.7 | ng/µL | 9.335 | 5.072 | 1.84 | 1.52 |
| S102 | Geldoc PC | 60.7 | ng/µL | 1.214 | 0.702 | 1.73 | 0.52 |
| S103 | Geldoc PC | 51.3 | ng/µL | 1.026 | 0.595 | 1.72 | 0.15 |
| S104 | Geldoc PC | 113.6 | ng/µL | 2.272 | 1.265 | 1.8 | 0.31 |
| S105 | Geldoc PC | 32.4 | ng/µL | 0.648 | 0.355 | 1.82 | 0.08 |
| S106 | Geldoc PC | 11.1 | ng/µL | 0.223 | 0.128 | 1.75 | 0.03 |
| S107 | Geldoc PC | 10.9 | ng/µL | 0.219 | 0.116 | 1.89 | 0.03 |
| S108 | Geldoc PC | 181.2 | ng/µL | 3.624 | 1.93 | 1.88 | 0.61 |
| S109 | Geldoc PC | 53.6 | ng/µL | 1.073 | 0.635 | 1.69 | 0.15 |
| S110 | Geldoc PC | 11.2 | ng/µL | 0.224 | 0.141 | 1.59 | 0.03 |
| S111 | Geldoc PC | 17.5 | ng/µL | 0.35 | 0.202 | 1.73 | 0.07 |
| S112 | Geldoc PC | 23 | ng/µL | 0.461 | 0.262 | 1.76 | 0.07 |
